# Psychotic‐like experiences and associated factors in resident physicians: A Canadian cross‐sectional study

**DOI:** 10.1111/eip.13564

**Published:** 2024-05-20

**Authors:** Vincent Paquin, Emilie Guay, Christophe Moderie, Camille Paradis, Nima Nahiddi, Frederick L. Philippe, Marie‐Claude Geoffroy

**Affiliations:** ^1^ Department of Psychiatry McGill University Montréal Quebec Canada; ^2^ Douglas Mental Health University Institute Montréal Quebec Canada; ^3^ Lady Davis Institute for Medical Research, Jewish General Hospital Montréal Quebec Canada; ^4^ Department of Psychiatry University of Toronto Toronto Ontario Canada; ^5^ Department of Psychology University of Québec in Montréal Montréal Quebec Canada

**Keywords:** discrimination, medical education, medical residents, psychosis, psychotic experiences

## Abstract

**Aim:**

Medical residency training is associated with a range of sociodemographic, lifestyle and mental health factors that may confer higher risk for psychotic‐like experiences (PLEs) in residents, yet little research has examined this question. Thus, we aimed to document the prevalence and associated factors of PLEs among resident physicians.

**Methods:**

Physicians enrolled in residency programmes in the Province of Québec, Canada (four universities) were recruited in Fall 2022 via their programme coordinators and social media. They completed an online questionnaire assessing PLEs in the past 3 months (the 15‐item Community Assessment of Psychic Experiences), as well as sociodemographic characteristics, lifestyle and mental health. Analyses included survey weights and gamma regressions.

**Results:**

The sample included 502 residents (mean age, 27.6 years; 65.9% women). Only 1.3% (95% CI: 0.5%, 4.0%) of residents met the screening cut‐off for psychotic disorder. Factors associated with higher scores for PLEs included racialised minority status (relative difference: +7.5%; 95% CI: +2.2%, +13.2%) and English versus French as preferred language (relative difference: +7.9% 95% CI: +3.1%, +12.9%), as well as each additional point on scales of depression (relative difference: +0.8%; 95% CI: +0.3%, +1.3%) and anxiety (relative difference: +1.3%; 95% CI: +0.8%, +1.7%). In secondary analyses, racialised minority status was associated with persecutory items, but not with other PLEs. Gender, residency programmes and lifestyle variables were not associated with PLEs.

**Conclusions:**

This study found low reports of PLEs in a sample of resident physicians. Associations of PLEs with minoritised status may reflect experiences of discrimination.

## 1 INTRODUCTION

In many countries including Canada and the United States, residency is the period of training that follows medical school. Resident physicians (sometimes referred to as interns, house officers or registrars as a function of the year of training) must complete full‐time clinical rotations and call duties over a period of two to 7 years before transitioning to independent practise. Residency training can be a challenging period for physicians, evident in the high prevalence of burn‐out, depression and anxiety (De Mélo Silva Júnior et al., 2022; Ferguson et al., 2020; Mata et al., 2015). Work‐related sleep constraints (Mansukhani et al., 2012), bullying and harassment (Ferguson et al., 2020) have been identified as potential contributors to mental health problems during residency.

Although previous studies have focused on adjustment and emotional difficulties in physicians such as burn‐out, depression and anxiety (Ferguson et al., 2020; Mata et al., 2015; Rodrigues et al., 2018), the spectrum of psychosis symptoms in this population remains underexplored. According to the psychosis spectrum model, psychotic symptoms emerge on a continuum of severity in the general population, with the mildest forms of psychosis phenomena (psychotic‐like experiences; PLEs) being the most common, and the more severe forms (clinical psychosis) being less common and more impairing (Guloksuz & Van Os, 2018; Staines et al., 2022). PLEs, such as paranoid ideas, bizarre experiences and quasi‐hallucinatory phenomena, affect up to 5%–7% of individuals in the community and are particularly common in young adults (Staines et al., 2022; Sullivan et al., 2020). Although in most cases PLEs do not progress to psychotic disorders, they indicate an increased risk of depression, anxiety, suicide attempts and other mental health problems (McGrath et al., 2016; Yates et al., 2019). PLEs are associated with cognitive biases, such as jumping to conclusion and belief inflexibility (Livet et al., 2020), and with lower cognitive performance and occupational functioning (Mollon et al., 2016; Owan et al., 2022).

Physicians face several potential risk factors for PLEs, including sleep disruptions, cannabis use, high levels of digital media use and low levels of physical activity (Barton et al., 2018; Paquin et al., 2024a, 2024b; Stubbs et al., 2017). Relative to attending physicians, residents may be particularly at risk given their younger age and potential exposure to bullying and discrimination, which are known risk factors for these experiences (Anglin, 2023; Pignon et al., 2021; Staines et al., 2023). Minority‐specific adversity, another factor associated with increased PLEs (Anglin, 2023; Anglin & Lui, 2023), may further compound the risk in residents from racialised minority groups or who speak a minority language. However, despite the potential relevance of PLEs for physician well‐being and functioning, the prevalence and correlates of these symptoms remain unknown. Such data could hypothetically help de‐stigmatize PLEs and better situate their clinical significance as markers of psychological distress within the medical profession. We therefore surveyed resident physicians in the province of Québec, Canada to estimate the prevalence of PLEs in the past 3 months, as well the associations of these experiences with sociodemographic characteristics, lifestyle factors, and symptoms of depression and anxiety.

## 2 METHODS

### 2.1 Study design

Eligible participants for this cross‐sectional study were resident physicians actively training in any residency programme in the Province of Québec, Canada. There were no exclusion criteria. Participants were recruited between September and November 2022 to complete an online questionnaire in either French or English. In 2022, the province counted 3966 residents across four universities: Laval University, McGill University, Sherbrooke University and University of Montréal. The study consent form and questionnaires are publicly accessible at https://osf.io/bavfj/. The study received ethical approval from the institutional review board of the University of Quebec in Montreal (#4552_e_2021).

For recruitment, we contacted the coordinators or administrators of all 60 residency disciplines across the four universities and asked them to distribute our study advertisement among their residents. Additionally, we circulated the study advertisement on social media residency‐related groups. Interested residents were then invited to consult the study website to electronically sign a consent form and complete the questionnaire. An optional random draw for 50 gift cards of 75 CAD was offered to encourage participation.

### 2.2 Measures

PLEs in the past 3 months were measured with the 15‐item Community Assessment of Psychic Experiences (CAPE) (Capra et al., 2017). The CAPE has been validated in French and English (Brenner et al., 2007) and shows good internal reliability and validity for detecting psychotic experiences (Bukenaite et al., 2017; Capra et al., 2013; Jaya et al., 2021). Its items (detailed in the Results) evaluate persecutory ideation, bizarre experiences, and perceptual abnormalities. Each item is rated on scale of 1 = ‘never’ to 4 = ‘nearly always’, and the global score is the average rating across all items (range: 1–4). A mean score of 1.75 on the longer 20‐item version of the CAPE has been proposed as a cut‐off to screen for psychotic disorders in the general population (Jaya et al., 2021). In accordance with the World Mental Health Composite International Diagnostic Interview (Kessler & Ustün, 2004) and a previous adaptation of the CAPE (Paquin et al., 2024a), we instructed participants to exclude experiences that occurred only while under the influence of alcohol, drugs, or medications that were not prescribed.

Sociodemographic characteristics included age, gender, residency (year of training, faculty of medicine and program) and racialised minority status (defined as Arab, Asian, Indigenous, Hispanic, Black or African American or Other; comparator: White). Preferred language was based on whether participants opted to complete the questionnaire in English or French. Digital media use was measured with 3 items adapted from the Coronavirus Health and Impact Survey (Nikolaidis et al., 2021) that evaluate daily time spent in the past 3 months on (1) TV or streaming platforms, (2) social media and (3) video games. Response options for each type of media included: ‘never/did not use’, ‘under 1 h’, ‘1–3 h’, ‘4–6 h’ and ‘more than 6 h’ per day. These categories were re‐coded as numeric values according to their midpoints and were summed across the 3 items to estimate total digital media use, to a maximum of 18 h/day. Cannabis use in the past 2 weeks was reported on a 4‐point scale (‘never’, ‘1–2 times per week’, ‘3 or more time per week’ and ‘everyday’) and was re‐coded dichotomously as 0 = never and 1 = ‘1–2 times per week’ or more. Sleep duration (h) was measured by assessing typical sleep habits on days with commitment such as work or school using the Munich Chronotype Questionnaire (Roenneberg et al., 2003). We also asked participants to estimate how many nights of sleep in the past month were significantly affected by clinical duties (1 item; range: 0–30 nights/month). Other measures included physical activity in metabolic equivalent task (MET)—minutes/week using the International Physical Activity Questionnaire—Short Form (Craig et al., 2003) and rescaled to mean = 0, SD = 1; symptoms of depression using the 9‐item Patient Health Questionnaire (range: 0–27) (Carballeira et al., 2007; Kroenke et al., 2001) and symptoms of anxiety using the 7‐item General Anxiety Disorder (range: 0–21) (Micoulaud‐Franchi et al., 2016; Spitzer et al., 2006).

### 2.3 Statistical analysis

Analyses were conducted in R version 4.1.2. Confidence intervals (CI) of 95% not overlapping the null were considered statistically significant. The prevalence of endorsing at least one PLE was estimated as the frequency among participants (95% CI) of scoring at least one item above ‘never’. Associations of sociodemographic characteristics, lifestyle and mental health measures with PLEs were examined using generalised linear models with gamma distributions and log‐link functions to accommodate the skewed distribution of PLEs (Ng & Cribbie, 2017). To adjust for differences between characteristics of our sample versus of the target population, we applied sample weights. Raking ratio estimation (Kalton & Flores‐Cervantes, 2003; Lumley, 2020) was applied to calibrate weights to gender, language, age, training faculty of medicine and program of training based on census data from the provincial physician organisation (Table S1; census data on racialised minority group was not available). Age and gender were additionally included as covariates in the regression models.

## 3 RESULTS

### 3.1 Sample characteristics

We recruited 564 participants across 42 programmes, representing 14.5% of the total population of 3966 resident physicians. The sample included 502 participants with complete data (mean [SD] age, 27.6 (3.64) years; 171 [34.1%] men). Tables 1 and 2 present sociodemographic, lifestyle and mental health characteristics in the sample. The sample and the total resident population were similar on gender, language and program of training. Differences of the sample versus population included younger age, over‐representation of residents from Laval University and under‐representation of residents from the University of Montreal (Table S1).

**TABLE 1:** Sociodemographic characteristics of study participants included in the analyses (*n* = 502).

	Mean (SD) or *N* (%)
Age in years	27.6 (3.64)
Gender	
Men	171 (34.1%)
Women	331 (65.9%)
Non‐binary or other gender	<5
Racialised or ethnic group	
Arab	40 (8.0%)
Asian	40 (8.0%)
Black or African American	<5
Hispanic	<5
Indigenous	<5
White	391 (77.9%)
Other	23 (4.6%)
Language	
English	93 (18.5%)
French	409 (81.5%)
Training faculty of medicine	
Laval University	208 (41.4%)
McGill University	124 (24.7%)
Sherbrooke University	138 (27.5%)
University of Montréal	32 (6.37%)
Program of training	
Family medicine	124 (24.7%)
Medical specialties	195 (38.8%)
Psychiatry	58 (11.6%)
Paediatric specialties	18 (3.6%)
Surgical specialties	65 (12.9%)
Other specialties	42 (8.4%)

*Note*: The category of non‐binary or other gender in the study sample was not included in the analyses due to small group size (*n* < 5) limiting statistical inference.

**TABLE 2:** Lifestyle and mental health characteristics of study participants (*n* = 502).

	Mean	SD	Q_1_, Q_3_
Digital media use, hours/day	3.72	3.31	2.00, 4.00
Nights affected by work, past month	5.46	5.43	2.00, 7.00
Sleep duration before workdays, hours	7.28	0.91	6.75, 7.90
Physical activity, metabolic equivalent task—minutes/week	1857	1634	720, 2452
Depression	5.19	4.51	2.00, 7.00
Anxiety	5.90	4.47	2.50, 8.00

	*N*	%
Cannabis use, past 2 weeks		
Any	29	5.8
None	473	94.2

*Note*: Q_1_, Q_3_, lower and upper quartiles. Depression: 9‐item Patient Health Questionnaire (range: 0–27). Anxiety: 7‐item General Anxiety Disorder (range: 0–21).

Abbreviation: SD, standard deviation.

### 3.2 Prevalence of PLEs

Table 3 presents the frequency of endorsement of each PLE item. Overall, the weighted 3 month prevalence of endorsing at least one PLE was 56.7% (95% CI: 48.9%, 64.0%), and the weighted prevalence of meeting the screening cut‐off for psychotic disorders was 1.3% (95% CI: 0.5%, 4.0%). The weighted prevalence of subtypes of PLEs was 54.7% (95% CI: 46.9%, 62.0%) for persecutory ideation, 10.8% (95% CI: 6.3%, 18.0%) for bizarre experiences and 1.2% (95% CI: 0.5%, 3.0%) for perceptual abnormalities. Prevalence estimates were similar without survey weights (Table S2).

**TABLE 3:** Psychotic‐like experience items in descending order of frequency by subscale.

Subscale	Item	Frequency
Persecutory ideation	Felt as if some people are not what they seem to be	32.1%
	Felt as if people seem to drop hints about you or say things with a double meaning	31.9%
	Felt that people look at you oddly because of your appearance	16.2%
	Felt that you are being persecuted in anyway	13.4%
	Felt as if there is a conspiracy against you	3.6%
Bizarre experiences	Felt as if electrical devices such as computers can influence the way you think	3.0%
	Felt as if the thoughts in your head are not your own	2.8%
	Heard your thoughts being echoed back at you	2.4%
	Your thoughts have been so vivid that you were worried other people would hear them	1.2%
	Felt as if you are under the control of some force or power other than yourself	<1.0%
	Felt as if the thoughts in your head are being taken away from you	<1.0%
	Felt as if a double has taken place of a family member, friend or acquaintance	<1.0%
Perceptual abnormalities	Heard voices when you are alone	1.0%
	Seen objects, people or animals that other people can't see	<1.0%
	Heard voices talking to each other when you are alone	<1.0%

*Note*: Unweighted frequency of single items, calculated as the proportion of participant ratings above the minimal category of ‘never’ on the 15‐item Community Assessment of Psychic Experiences.

### 3.3 Correlates of PLEs

**FIGURE 1:**
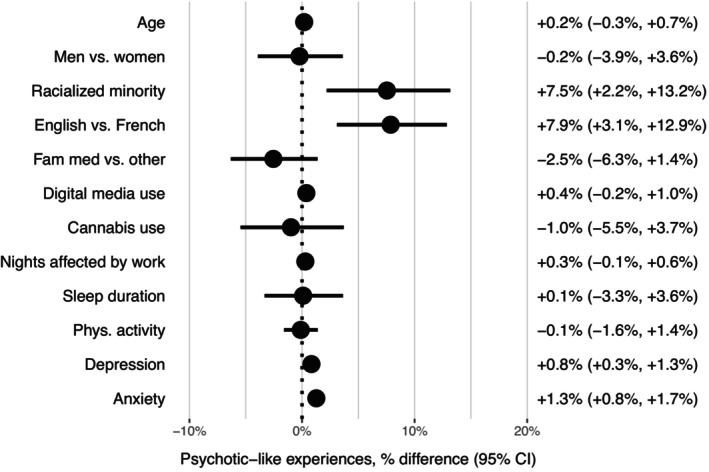
Associations of sociodemographic characteristics, lifestyle, and mental health with psychotic‐like experiences. Generalized linear models with gamma distributions and log‐link functions, adjusted for age and gender and with survey weights (*n* = 502). Age: in years. Racialised minority: any ethnoracial category other than White. Program of training: dichotomised as family medicine versus other programmes. Digital media use: hours/day. Cannabis use: any versus none in the past 2 weeks. Nights affected by work: in the past month. Phys. activity: metabolic equivalent task (MET)–minutes/week, rescaled to mean = 0, SD = 1. Depression: 9‐item Patient Health Questionnaire (range: 0–27). Anxiety: 7‐item General Anxiety Disorder (range: 0–21).

Associations of sociodemographic characteristics, lifestyle and mental health measures with PLEs are presented in Figure 1. Racialised minority status and preferred language of English were associated with higher levels of PLEs. Each additional point on the depression and anxiety scales were also associated with higher PLEs. Other factors were not significantly associated with PLEs (Figure 1), including subcategories of training programmes (medical specialties, psychiatry, paediatric specialties, surgical specialties; data not shown). Without survey weights (Figure S1), the findings were similar, with additional weak associations of higher digital media use (+0.5%; 95% CI: +0.2%, +0.8%) and more nights affected by work (+0.3%; 95% CI: +0.1%, +0.5%) with more PLEs, and an association of longer sleep duration with fewer PLEs (−1.3%; 95% CI: −2.5%, −0.1%).

Post‐hoc, we examined whether racialised minority status and preferred language were independently associated with PLEs after adjusting for each other, given potential covariance between the two factors. We again included age and gender as covariates, as well as training faculty of medicine to account for university‐related sociodemographic differences. Racialised minority status (relative difference: +5.6%, 95% CI: +1.1%, +10.3%) and English versus French (+7.0%, 95% CI: +2.8%, +11.4%) remained significantly associated with higher levels of PLEs, whereas faculty of medicine was not.

Next, we considered that higher endorsement of persecutory ideation among minority residents could reflect experiences of discrimination not related to psychosis spectrum phenomena. To explore this, we separately tested associations of racialised minority and language with two secondary indices of PLEs: persecutory ideation only (5 items), and other PLEs (bizarre experiences and perceptual abnormalities; 10 items). We combined the latter two subscales given their lower prevalence. Racialised minority (+14.8%; 95% CI: +5.0%, +25.5%) and English versus French (+15.1%; 95% CI: +6.3%, +24.7%) were significantly associated with higher levels of persecutory ideation. Racialised minority was not significantly associated with other PLEs (+3.2%; 95% CI: −0.6%, +7.1%), but English versus French was significantly associated with higher levels thereof (+3.7%; 95% CI: +0.2%, +7.2%).

## 4 DISCUSSION

In a large sample of Canadian resident physicians, we found that persecutory ideation was the most common type of PLEs, and that higher levels of PLEs were associated with racialised minority status, selecting English versus French as preferred language, and higher levels of depression and anxiety. To our knowledge, this is the first study examining the prevalence and correlates of PLEs in physicians.

Although more than half of residents reported at least one type of PLE in the past 3 months, only 1.3% met the screening cut‐off for psychotic disorder. These results are lower than those found in samples from the general population: for example, in a sample of 425 young adults from the general population of Québec (median age, 22 years; 82.5% female), 81.3% of participants endorsed at least one item on the same version of the CAPE as used in the present study, and 10.4% met the screening cut‐off for psychotic disorders (Paquin et al., 2024a). Across stages of medical training, there is limited data on the prevalence of PLEs. A survey of 639 Indonesian medical students found significant levels of psychoticism and paranoid ideations in 1.7% and 2.2% of respondents, respectively, based on the subscales of the Symptom Checklist‐90 and their *T*‐score thresholds (Siste et al., 2021). Of note, however, students with severe psychotic disorders or substance use were excluded from that study. In a retrospective study of medical records of 335 resident physicians admitted to a specialised mental health service in Spain between 1998 and 2018, 45 (2.4%) residents had a diagnosis of schizophrenia or other psychotic disorder, whereas adjustment and affective disorders were the most common diagnoses (Braquehais et al., 2021). Individuals with high levels of psychosis propensity may be less likely to enter medical school or residency because of the impacts of psychosis on academic performance, stigma and socio‐economic disadvantage, potentially explaining the lower prevalence of psychosis spectrum outcomes among residents. Stigma and desirability bias may also lead residents to under‐report PLEs.

Racialised minority group and selecting English (a minority language in Québec) versus French were associated with higher levels of PLEs. To some extent, these results may reflect the influence of cultural and language‐related factors on the experience, interpretation and social acceptability of questionnaire items. Although the CAPE has been validated in multiple languages (including French and English), a study in the Netherlands, Nigeria and Norway identified potential cross‐cultural variations in the questionnaire properties, with higher prevalence of persecutory and bizarre experiences in the Nigerian versus other cohorts (Vermeiden et al., 2019). Importantly, higher prevalence of PLEs among minoritised residents may also reflect experiences of discrimination. In a survey of 833 resident physicians across Canada, 78.2% reported experiencing harassment or intimidation in the past year, with culture (10.5%), ethnicity (9.4%) and language (1.5%) cited among perceived bases for these experiences (Resident Doctors of Canada, 2018). Patients were identified as the most common source of harassment or intimidation, whereas other frequently cited sources included physicians, co‐residents and allied health professionals. Thus, it is possible that the present associations of racialised and linguistic minority groups with PLEs reflect the impact of discrimination at work, which could also explain the observed association of PLEs with depression and anxiety. It should be noted, however, that the racialised and linguistic minority categories aggregate various backgrounds which likely come with heterogeneous experiences, such that, for example, two individuals from different racialised groups do not encounter the same degrees and forms of discrimination. Other sources of adversity (e.g., related to housing, police surveillance or adaptation to a different country and health systems among international medical graduates; Anglin, 2023; Murillo Zepeda et al., 2022) could further contribute to increased levels of persecution reported on the PLE questionnaire.

When examining a modified score for PLEs without persecutory ideation items, racialised minority status was no longer significantly associated with PLEs. Modifying the score in this way may have constrained the statistical power to detect group differences, since bizarre experiences and perceptual abnormalities were less prevalent than persecutory ideas. Additionally, it seems likely that the CAPE primarily captured thoughts and feelings stemming from lived experience of discrimination, rather than ‘true’ psychosis spectrum phenomena, in residents from racialised minority groups. Determining whether group differences in PLEs are accurate or if they reflect issues of measurement validity is challenging. In clinical and community‐based cohorts, many studies have shown that racialised minority status is associated with higher risk of PLEs and psychotic disorders, with evidence that interpersonal discrimination and broader socio‐environmental structures of disadvantage may underlie this excess of risk (Anglin, 2023; Anglin et al., 2021; DeVylder et al., 2023). For example, in a sample of 955 college students in the United States, Anglin and Lui (2023) evaluated PLEs using the Prodromal Questionnaire–Likert and found that Black participants reported higher levels of these experiences than White and Latinx participants. Higher levels of PLEs among Black people were found in the sub‐domains of paranoia, suspiciousness and unusual thinking, and this excess in PLEs was significantly explained by greater exposure to racial microaggressions and major racial discriminatory experiences. The studies by Anglin and Lui (2023) and others (Anglin et al., 2021; DeVylder et al., 2023) thus suggest that discrimination as a pathogenic mechanism may directly contribute to the risk of PLEs. However, given the low prevalence of non‐persecutory PLEs in the present sample, and the lack of prospective data on potential mediators (e.g., microaggressions and discriminatory experiences), we could not examine the role of discrimination on PLEs among medical residents.

We did not find robust associations between other sociodemographic and lifestyle factors and PLEs. There was no significant gender difference in levels of PLEs in our sample, in line with the lack of consistent gender patterning of PLEs in the general population (Staines et al., 2023). Lifestyle factors such as higher levels of digital media use, sleep disruptions and cannabis use have been identified as correlates of PLEs in the general population (Barton et al., 2018; Paquin et al., 2024a), lower levels of physical activity have been cross‐sectionally associated with greater likelihood of psychotic disorders (Stubbs et al., 2017), and these factors have also been linked to poorer mental health in resident physicians (Mansukhani et al., 2012; Mathew et al., 2022; Ueno et al., 2020; Weight et al., 2013). In our study, higher digital media use and poorer sleep were weakly associated with more PLEs, but these associations were not robust to adjustment with survey weights. Relatively homogeneous population characteristics, including a skewed distribution of PLEs and infrequent reports of cannabis use, may have constrained the statistical power to detect associations of smaller magnitude, possibly explaining some of these negative findings.

In light of the above, we believe a few improvements can be made in future research on PLEs in resident physicians. For one thing, to better interpret the significance of minority‐specific differences in PLE scores among medical residents, data on discriminatory experiences should be included. Another issue to consider is the choice of scale: having a measure of PLEs with greater sensitivity for detecting non‐persecutory experiences, perhaps such as the Prodromal Questionnaire, may help detect associations between PLEs and social adversity that are not bound to threat‐related perception. Lastly, the inclusion of participatory methods and qualitative data will enable a better representation of the diverse perspectives and experiences of resident physicians, ultimately contributing to a greater understanding of the interplay of social adversity, mental health and PLEs in residents.

### 4.1 Limitations and future directions

This study is unique in its examination of PLEs in a large sample of residents from various training stages and programmes and recruited from a well‐characterized target population. The response rate was low and, despite the inclusion of survey weights, sampling bias may have occurred if residents were more or less likely to participate as a function of their mental health, work schedules, economic status or other factors relevant to the associations examined here. Other factors, such as social desirability, may have led participants to under‐report their PLEs and lifestyle habits such as cannabis use. Self‐reporting of PLEs may also be less specific than clinician‐rated assessments, with items potentially tapping into other domains such as anxiety or social adversity; however, both self‐reported and clinician‐rated psychotic experiences are associated with the risk of psychotic disorders and with environmental risk factors for psychosis, supporting their convergent validity (Pries et al., 2018; Staines et al., 2022; Werbeloff et al., 2012).

In conclusion, this study provides novel data on the prevalence and correlates of PLEs in medical residents, finding that these experiences were not commonly reported, and that they were associated with depression, anxiety, and racialised and linguistic minority status. The association of racialised minority status with PLEs was specific to persecutory items, suggesting it was more a reflection of discriminatory experiences than of psychosis spectrum phenomena. Additional measures should be included in future research to further contextualise and expand on the present findings, including of discrimination and social adversity, other training‐related factors such as program support and access to mental health care, and other forms of minoritised identities such as sexual orientation, gender identity and lower socio‐economic status. Anti‐discrimination training curricula for trainees and staff, and faculty‐funded mentorship from staff to residents of minoritised groups, are examples of interventions that may help counter the impacts of racism and discrimination on resident well‐being (Jarvis et al., 2023). Although more research is needed to situate the significance of PLEs among other indicators of mental health in resident physicians, opening the discussion holds the potential to reduce stigma around PLEs within the medical profession and to promote institutional action on the sources of adversity that engender them.

## FUNDING INFORMATION

This study was supported by research funding from the Fédération des médecins résidents du Québec (FMRQ) awarded to VP, FLP and MCG. VP is supported by an award from the Fonds de recherche du Québec–Santé and the Ministère de la santé et des services sociaux (FRQS‐MSSS).

## CONFLICT OF INTEREST STATEMENT

This study was supported by research funding from the Fédération des médecins résidents du Québec (FMRQ) awarded to VP, FLP and MCG. The FMRQ, which is the professional union for resident physicians in the province of Québec, Canada, had no role in study design, data collection and analysis, decision to publish, or preparation of the manuscript.

## Supporting information


**Data S1.** Supporting information.

## Data Availability

Data are not publicly available due to their containing information that could compromise the privacy of research participants.
