# Supplementary material for: Psychotic‐like experiences and associated factors in resident physicians: A Canadian cross‐sectional study
Source: Early Interv Psychiatry. 2024 May 20;19(1):e13564. doi: 10.1111/eip.13564 (PMC11730528; doi:10.1111/eip.13564)
Supplement: Supplementary file 1 — Data S1. Supporting information. [file EIP-19-0-s001.docx]

**Table S1. Characteristics of the sample and of the target population used for raking ratio estimation**

| **Characteristic** | **Sample, N=502** | **All residents in Québec, N=3966** |
| --- | --- | --- |
| Age in years |  |  |
| <27 | 43.8% | 14.6% |
| 27 to <29 | 28.7% | 28.1% |
| ≥29 | 27.5% | 57.2% |
| Gender |  |  |
| Men | 34.1% | 43.9% |
| Women | 65.9% | 56.1% |
| Language |  |  |
| English | 18.5% | 28.6% |
| French | 81.5% | 71.4% |
| Training faculty of medicine |  |  |
| Laval University | 41.4% | 21.1% |
| McGill University | 24.7% | 31.9% |
| Sherbrooke University | 27.5% | 15.6% |
| University of Montréal | 6.4% | 31.4% |
| Program of training |  |  |
| Family medicine | 24.7% | 25.3% |
| Other programs | 75.3% | 74.7% |

Data on the total resident population in 2022 was provided to the authors by the Collège des médecins du Québec ([www.cmq.org](http://www.cmq.org)). Age in the total population was available as a mean (mean age, 29.8 years) and was converted to categorical format based on the distribution of age in the study sample. Survey weights were estimated using the raking ratio method.

**Table S2. Prevalence of psychotic-like experiences without and with survey weights (n=502)**

| **Outcome** | **Frequency, unweighted** | **Prevalence estimate, weighted (95% CI)** |
| --- | --- | --- |
| Psychotic-like experience, any | 52.4% | 56.7% (48.9%, 64.0%) |
| Screening cut-off for psychotic disorder | 0.8% | 1.3% (0.5%, 4.0%) |
| Persecutory ideation, any | 51.4% | 54.7% (46.9%, 62.0%) |
| Bizarre experiences, any | 7.8% | 10.8% (6.3%, 18.0%) |
| Perceptual abnormalities, any | 1.2% | 1.2% (0.5%, 3.0%) |

**Figure S1. Unweighted** **associations of sociodemographic characteristics, lifestyle, and mental health with psychotic-like experiences**

Generalised linear models with Gamma distributions and log-link functions, adjusted for age and gender, without survey weights (n=502).
